# Supplementary material for: Conversion of Pulse Protein Foam-Templated Oleogels into Oleofoams for Improved Baking Application
Source: Foods. 2022 Sep 17;11(18):2887. doi: 10.3390/foods11182887 (PMC9498664; doi:10.3390/foods11182887)
Supplement: Supplementary file 1 [file foods-11-02887-s001.zip › foods-1844409-supplementary.pdf]

Supplementary data

Conversion of pulse protein foam-templated oleogels into oleofoams for improved baking application

Athira Mohanan, Kim Harrison, David M. L. Cooper, Michael T. Nickerson and Supratim Ghosh*

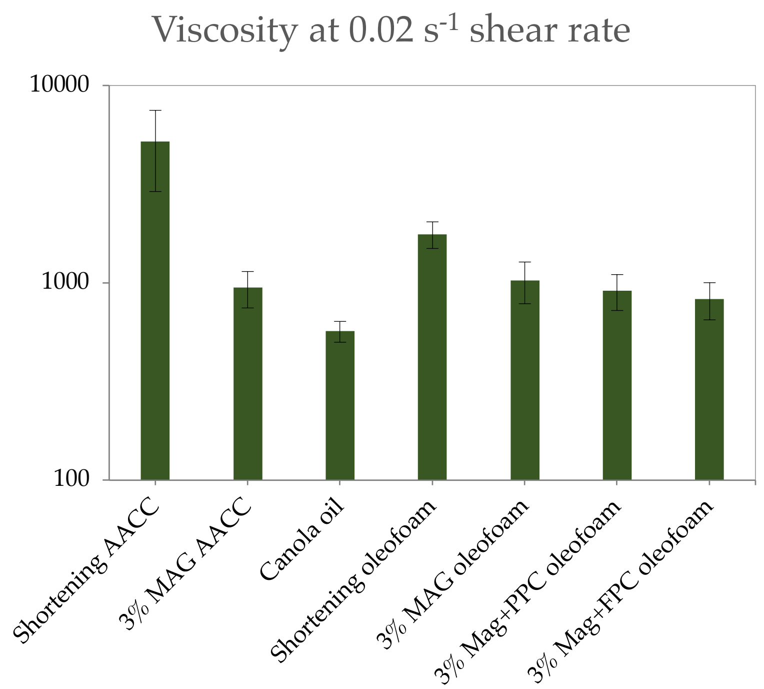

Figure S1. Comparison of low-shear viscosity of different cake batters at 0.015 s-1 shear rate. Shortening-AACC and 3%MAG AACC batters were produced using the AACC method. All the other batters, prepared with canola oil or oleofoams, used the new method developed in the lab. MAG: monoacylglycerols, PPC: pea protein concentrate-stabilized foam, FPC: faba bean protein concentrate-stabilized foam.
